# Students’ cross-domain mindset profiles and academic achievement in Finnish lower-secondary education

**DOI:** 10.3389/fpsyg.2025.1514879

**Published:** 2025-01-23

**Authors:** Jenni Laurell, Ita Puusepp, Kai Hakkarainen, Kirsi Tirri

**Affiliations:** Faculty of Educational Sciences, University of Helsinki, Helsinki, Finland

**Keywords:** mindsets, domain-specificity, latent profile analysis, creativity, lower-secondary education

## Abstract

**Introduction:**

This study uses a person-centered approach to explore Finnish lower-secondary school students’ (*N* = 1106) mindsets across intelligence, giftedness, and creativity. It further investigates the relationship between mindsets profiles, school achievement in various subjects, and gender differences, aiming to address the domain-specificity of the three ability domains.

**Methods:**

A self-reported questionnaire was used to measure students’ mindsets, with latent profile analysis (LPA) identifying distinct profiles. School achievement was assessed through academic grades in core and arts subjects, while gender differences in profile membership were examined via logistic regression.

**Results:**

Four mindset profiles emerged: *Growth, Fixed, Mixed*, and *Opposing*. Most students exhibited consistent “general” mindsets across domains, except those in the Opposing profile, who combined a growth mindset for intelligence and creativity with a fixed mindset for giftedness. Students in the Opposing profile outperformed others in mathematics and foreign languages, while those in the Growth profile excelled across other subjects. The Fixed profile was linked to the lowest achievement, except in reading, foreign languages, and music, where Mixed and Fixed profiles performed similarly. Girls were more likely to belong to the Growth profile, while boys dominated the Fixed and Opposing profiles.

**Discussion:**

The findings highlight the cross-domain nature of mindsets but reveal unique domain-specific variations, particularly for giftedness. These differences influenced academic outcomes, underscoring the nuanced role of mindsets in student achievement. Gender disparities in mindset profiles align with observed differences in school performance.

**Conclusion:**

By identifying distinct mindset profiles, this study emphasizes the complexity of students’ beliefs and possible educational implications. Future research should explore qualitative aspects of mindset formation across ability-related constructs, its broader motivational frameworks, and their relation to students’ academic outcomes.

## Introduction

People’s beliefs about the malleability of human qualities are referred to as mindsets. Mindsets reflect how individuals view the nature of human attributes, such as intelligence and personality, as either malleable and incremental or fixed and static (Dweck, 2017, p. 6). These mindsets are also termed implicit theories or beliefs. Mindsets exist along a spectrum ranging from growth to fixed. A growth mindset (an incremental view of human qualities) refers to the belief that human characteristics can be developed through effort and persistence. In contrast, a fixed mindset (an entity view of human qualities) reflects the belief that these characteristics are stable and unchangeable. A substantial body of research has explored how students’ mindsets about intelligence explain differences in students’ goals and behavior in education. Many studies have results suggesting that holding an incremental (growth) view of intelligence supports students’ learning motivation (e.g., Burnette et al., 2013; Dweck and Yeager, 2019; Rhew et al., 2018), leads to higher grades (Blackwell et al., 2007; OECD, 2018; Paunesku et al., 2015), and fosters greater academic aspirations (Yeager et al., 2019). Nevertheless, investigating students’ implicit beliefs beyond intelligence is essential because those may vary across attributes. In other words, individuals can hold differing implicit beliefs about various characteristics, such as intelligence, giftedness, creativity, or personality traits. Additionally, mindsets are found to be domain-specific (Dweck and Molden, 2017, p. 136), which adds to the need to investigate mindsets across domains.

Psychological constructs such as intelligence, giftedness, and creativity are complex, and no single theoretical conception exists. Researchers have, for example, debated the conceptualization of giftedness for a century without attaining a unanimous result on its definition. The construct still heavily carries its historical roots and is easily associated with high intellectual ability—especially in laypeople’s’ everyday conversations. However, looking at just students’ high intellectual ability or academic skills, in general, is an exceptionally narrow way to view giftedness, as scholars today agree that giftedness can emerge in an extensive range of skills (Sternberg and Ambrose, 2021, pp. 513–515). Regarding mindsets about giftedness, Dweck (2000, p. 122) suggested that due to the word’s connotation, giftedness is likely viewed as a fixed entity as the word “gift” implies that no effort is required, and that giftedness bestows upon rare or fortunate individuals. A few studies have compared among varying-aged students, how their mindsets about intelligence and giftedness differ and how students’ mindset affects their achievement at school (Makel et al., 2015; Kuusisto et al., 2017; Laurell et al., 2022). As Dweck suggested, the findings of all three studies indicate that students perceive intelligence as a more malleable human quality than giftedness.

Creativity is increasingly recognized as a crucial characteristic of 21st-century learners (Binkley et al., 2011; OECD, 2019). Notwithstanding, to our knowledge, no studies have investigated mindsets about creativity, though we are aware of studies, e.g., Karwowski (2014) and Karwowski et al. (2017) that explored implicit beliefs about creativity, mainly in the creative fields and primarily focused on capturing the multidimensional nature of creativity by developing a new scale to measure its multidimensionality. Instead, we employed a commonly used scale to investigate implicit beliefs about creativity’s developmental or innate nature alongside intelligence and giftedness (Dweck, 2000). In this study, we simultaneously examine mindsets in intelligence, giftedness, and creativity, intending to understand the domain-specificity of these intertwined and overlapping constructs (see Kaufman and Sternberg, 2008, p. 71–83). Moreover, we aim to contribute to prior mindset research by adopting a person-centered approach to investigate students’ mindsets about intelligence, giftedness, and creativity. Additionally, we assess how students’ profile group membership relates to academic achievement in various subjects and gender. We chose the person-centered method as it provides a better understanding of mindsets’ context specificity and replicability. Acknowledging this is relevant as mindsets might be context-dependable constructs and more diverse and complex than initially theorized (Altikulaç et al., 2024). A person-centered method also identifies individuals who share similar features and classifies them into more homogeneous subgroups. The method enables the investigation of the characteristics and percentages of learners who respond inconsistently with theoretical expectations (Muthén and Muthén, 2000). Research about mindsets has mainly focused on analyzing whole-sample averages, and only a minority of mindset studies have thus far used a person-oriented approach, which does not assume homogeneity across the entire sample.

### Domain-specificity of mindset beliefs

In the early years of mindset theory development, Dweck et al. (1995) suggested that individuals can hold different mindsets about various attributes at once. For example, individuals might believe they can develop their intelligence but not their personality. In this case, a growth mindset provides a framework for organizing thoughts and guiding actions related to intelligence. In contrast, a fixed mindset shapes individuals’ thoughts and actions within the personality domain, reflecting the belief that personal traits, such as temperament, are static and unchangeable. Dweck et al. (1995) also proposed that some individuals might possess more generalized mindset beliefs across multiple attributes. Even if this is the case, investigating the domain-specificity or generality of implicit theories remains relevant, as perceptions about one attribute’s developmental or static nature do not necessarily imply that this perception extends to all attributes (O’Keefe et al., 2018).

Lewis et al. (2021) recently investigated adults’ global and domain-specific mindsets (e.g., personality, intelligence, math, writing) using a bifactor model. They explored the strength of generalized beliefs across domains and discovered that mindsets remained consistent across domains throughout the sample. The researchers emphasized that when multiple domains are not assessed simultaneously, correlations between separate domains may be overlooked, leading to missed insights. Furthermore, they suggested that simultaneous examination of multiple domains may help validate the assumption that mindsets specific to a particular domain are most relevant to outcomes related to that domain. They also propose that the significance of general mindset beliefs may vary depending on the context.

In another study implementing the bifactor model, Petscher et al. (2017) evaluated the dimensionality of general and reading-specific mindsets among fourth-grade students. They found a general growth mindset factor and specific aspects of general and reading-specific mindsets. These findings suggest that while individuals’ mindsets across multiple domains are likely to be related, mindsets remain distinct in different areas. Also, Schroder et al. (2016) found evidence that among university students, mental-health-related mindsets were simultaneously domain-specific (e.g., students’ depression mindsets predicted symptoms of depression) and general (e.g., anxiety mindset and general mindset factors predicted most symptoms). Yu and McLellan (2020) adopted a person-centered method to investigate the coherence of mindsets about intelligence and associated motivational constructs and how they functioned together and influenced adolescent student achievement in math and reading. In addition to the four student profiles discovered, they found evidence supporting the domain specificity of the motivational frameworks, as only 64% of students remained in the same profile across the two academic subjects. As these studies have demonstrated, individuals’ implicit beliefs are not straightforward, and those should be investigated in terms of the generality and specificity of mindsets and various contexts and circumstances.

### The relationship between mindsets in learning and academic achievement

Mindsets about intelligence have been widely studied to understand their influence on academic achievement (e.g., Yeager and Dweck, 2020). Studies included in a review article by Zhang et al. (2017) that investigated students’ mindsets and academic achievement demonstrated that growth mindsets about intelligence positively influenced academic achievement. However, some studies have failed to find an association between a growth mindset about intelligence and higher academic grades (e.g., Leondari and Gialamas, 2002). When mindsets are studied to understand their effect on achievement at school, mathematics is commonly included in the measures; Math is a focal academic subject widely regarded as more dependent on inherent cognitive abilities (Costa and Faria, 2018). Additionally, the relationship between a growth mindset and higher academic achievement is particularly evident in subjects like math because math often presents cumulative challenges that require sustained effort and adaptive motivational frameworks to overcome (Gunderson et al., 2018). Blackwell et al. (2007) revealed an upward trajectory in math grades over 2 years among students with a growth mindset about intelligence, while a belief that intelligence is fixed predicted a flat trajectory in students’ math grades. Moreover, in a study by Romero et al. (2014), students who endorsed a growth mindset about intelligence earned higher grades and were likelier to participate in advanced math courses over time. Kuusisto et al. (2017) found that comprehensive school students’ growth mindset about intelligence and fixed mindset about giftedness indicated higher math grades. Recent research has focused on students’ subject-specific mindsets (e.g., mathematics: Puusepp et al., 2023; reading: Petscher et al., 2017; language learning: Lou and Noels, 2019) and their influence on grades in specific subjects. The results of these studies demonstrate that a general mindset about intelligence does not predict subject-specific achievement as consistently as subject-specific mindsets (e.g., math-ability mindset, reading-ability mindset, and language-learning mindset).

Due to the somewhat conflicting findings and critique of the mindset theory, Yeager and Dweck (2020) have tempered expectations about the direct effects of mindsets on academic achievement, noting that an individual’s mindset does not affect academic achievement per se. Indeed, Rattan et al. (2015) argue that growth-minded students tend to earn better academic grades because the mindset is embodied in responses to setbacks in challenging learning situations. According to Barger et al. (2022), a growth mindset is not only about working hard but efficiently, acquiring, and using help and different resources. More specifically, it is not enough to believe that improvement is generally possible; it is vital to understand that effort is necessary and to have effective strategies. If these aspects are not internalized, continuing challenges might undermine an individual’s motivation just as much as believing their ability is fixed.

When investigating gender and mindsets, a meta-analysis by Butler (2014) found gender differences to be expected in the results of motivational studies. Regarding mindsets about intelligence, the findings are somewhat contradictory. While some studies (Spinath et al., 2003) have suggested that females are more likely than males to exhibit a growth mindset, Diseth et al. (2014) found that girls held a weaker growth mindset than boys. Using latent-profile analysis, Yu and McLellan (2020) revealed variations in the number and types of gendered mindset profiles (including a mindset with associated motivational constructs), with boys more often in profiles with a fixed mindset, which facilitated mastery goal pursuit (Ability-Focused and Disengaged). They suggested that the mindset itself, as a single variable, does not cause gender differences; instead, gender differences commonly arise when academic subject domains (e.g., math) are investigated alongside mindsets.

### The present study

As presented in the theoretical section, several studies have demonstrated that mindsets are not straightforward. It has been stated that the developmental or static nature of mindsets about abilities should be investigated in various contexts and circumstances to understand their generality, specificity, and relationship to students’ achievement in formal education. Our interest was in examining more than one mindset domain at a time (see Lewis et al., 2021) and an aim to enable the comparison of the findings of this study with previous international and domestic studies about intelligence and giftedness-related mindsets in formal schooling (Makel et al., 2015; Kuusisto et al., 2017; Laurell et al., 2022). Thus, using a person-centered approach, this study simultaneously examines students’ implicit beliefs, i.e., their mindsets across concepts of intelligence, giftedness, and creativity—Additionally, it investigates the relationship between profile group membership and academic achievement in various school subjects, as well as emerging gender differences asking the following questions:

1. What kinds of student profiles can be identified based on mindsets in the three domains?
2. How do the profile groups differ in (a) academic achievement and (b) gender?

### Context

Comprehensive education in Finland comprises primary school (grades 1–6, 7–12-year-old) and lower secondary school (grades 7–9, 13–16-year-old), followed by general upper secondary school (academic track) or vocational upper secondary school (vocational track), with the application process based on students’ cumulative GPA at the end lower secondary school.

The Finnish school system is considered as egalitarian, and inclusive, and students are supported individually based on their needs. Mandatory formal education is free of charge and the same for all students, without ability grouping. Nevertheless, schools today are increasingly segregated by socioeconomic status, especially in the Helsinki metropolitan area (Bernelius and Vaattovaara, 2016). The Finnish National Core Curriculum (NCC) (Finnish National Agency for Education, 2014) defines the educational goals for compulsory education. The highest-level aim is to encourage students’ academic performance by creating an inclusive learning environment that supports holistic psychosocial development alongside traditional cognitive abilities.

The current NCC places a strong emphasis on teaching future skills, which include an open-minded attitude and a growth mindset toward learning, acquiring knowledge across various academic domains, and being able to challenge oneself while studying, not to forget creative thinking that is highlighted as a teaching and learning objective in the NCC (Finnish National Agency for Education, 2014). The Finnish educational system employs a differentiation approach to identify gaps between students’ knowledge and the curriculum content (Laine and Tirri, 2021). In some cases, the lack of recognition at school can prevent students who exceed the objectives of NCC or are in some other way from fulfilling their educational potential. Mindset research conducted in Finland has reported gender differences in students’ mindsets, with Finnish boys displaying a stronger tendency toward a fixed giftedness mindset than girls but sharing similar mindsets about intelligence to their female counterparts (Kuusisto et al., 2017). Investigating gender differences is relevant because educational achievement in Finland is increasingly polarized by gender (Hautamäki et al., 2015; OECD, 2019).

## Materials and methods

### Procedure

The current study was part of a longitudinal research project: Growing Mind—Educational Transformations for Facilitating Sustainable Personal, Social, and Institutional Renewal in the Digital Age. The project arranged a data collection in Helsinki, Finland. The present study was included in the project’s ethical review, which was accepted by The University of Helsinki’s Research Ethics Committee and the municipality. Participation to the data collection was voluntary for the students, and for the schools. In total, 32 schools participated in the project’s data collection, with 3,262 ninth-grade students. As the participants were underage, consent was requested from their guardians in advance, and in total, 1,971 guardians gave consent to use their wards’ answers for research purposes.

The data used in this study was collected during regular school lessons in the fall semester of 2021. Teachers collaborated with researchers to initiate the data collection through an electronic survey using Qualtrics software. The questionnaire was completed on laptops or tablets provided by the school. At the beginning of the data collection, a short instructional video created by researchers from the project was shown to the participants to inform them about the research in general and its aims. The participants were informed about their right to withdraw from the process at any time, and permission to use their responses for research purposes was requested in writing before the commencement of the actual survey. The data collection procedure lasted an average of 35 min, and the extensive research survey (190 variables) took 20–25 min to complete.

The original raw data included 1,443 study participants. However, this dataset was cleaned from unreliable answers that would distort the results. The raw data included many questionable cases (empty, fake, untraceable names). For reliability, data was deleted if the participant (1) had answered jokingly, (2) had not answered more than 6% of the questionnaire, (3) had answered twice, (4) took part in the questionnaire without the permission of the guardian, or (5) did not permit to use their answer for research purposes. At the beginning of the analysis for the present study, the dataset included 1,260 participants. However, some participants (*n* = 154) quit the survey before reaching the section where the mindsets were evaluated. Thus, before the main analysis for this study, 154 cases were eliminated.

### Participants

A total of 1,106 participants (15–16 years old) were included in the main analysis for this study. The respondents were required to identify their gender at the beginning of the questionnaire, and 51.3% identified themselves as girls (*n* = 567) and 43.4% as boys (*n* = 480). In turn, 5.3% identified themselves as “other” or did not report their gender (*n* = 59).

### Mindset measures

Intelligence, giftedness, and creativity mindsets were assessed using the Implicit Theories of Intelligence Scale (ITI; Dweck, 2000). Within the framework of Dweck’s theory, we employed an instrument that intentionally refrains from providing respondents with predefined definitions of the constructs under investigation, meaning no explicit definitions of intelligence, giftedness, and creativity were given to participants in the questionnaire. The ITI scale originally consists of four entity statements and four incremental statements, but it was suggested by Dweck (2008) that the growth mindset items be omitted and only the fixed mindset items be used, as the growth mindset items can lead to a social desirability bias. In our survey, the scale consisted of Dweck’s three entity statements: “People have a certain amount of intelligence, and not much can be done to change it,” “To be honest, you cannot really change how intelligent you are,” and “People can learn new things, but cannot really change their basic intelligence,.” Scale was adapted to other domains by replacing “intelligence” with “giftedness,” and “creativity” which is a common manner in mindset-domain research (Burnette et al., 2013; Chiu et al., 1997). Each item was assessed with a 6-point scale from 1 (strongly agree) to 6 (strongly disagree), with higher scores indicating a greater endorsement of a growth mindset. The internal consistencies of the mindset scales were found to be good: Cronbach’s alpha for the intelligence scale was *α* = 0.89; for giftedness, it was *α* = 0.93, and for creativity, it was *α* = 0.94. Three mean scores were used.

### Academic achievement

Data regarding the grades was obtained from school year reports requested from the National Agency for Education at the end of the 2022 academic year. Academic grades in Finland range from 4 (lowest) to 10 (highest) and are based on teachers’ evaluations of tests, homework, classroom participation, and student effort (Finnish National Agency for Education, 2014). Instead of using the GPA of the school year reports, we evaluated grades in specifically chosen academic subjects: mathematics, reading, 1st compulsory foreign language (e.g., English, French, German), music, visual arts, and crafts.

### Data analysis

Confirmatory factor analysis (CFA) in R (Version 4.3.3) with the RStudio interface (Version 2024.04.1) and *lavaan* package (Version 0.6–17; Rosseel, 2012) was first conducted to determine the factor structure of the mindset measures. Subsequently, latent profile analysis (LPA) with the mean scores of the three mindset domains as indicator variables was performed in Mplus version 8.9 to explore the profile groups. The specification “TYPE = COMPLEX” with “school” as the cluster was applied to account for the nesting of students within schools (Muthen and Muthen, 2024). Solutions with 2–10 profiles were explored. The best solution was determined by considering theoretical interpretability, profile sample sizes, and the following fit indices: AIC, sample-size adjusted BIC (SABIC), entropy, and values of VLMR test. Smaller AIC and SABIC values indicate a better fit, higher entropy indicates greater classification certainty (with values larger than 0.80 indicating a “good” classification), while a non-significant VLMR test suggests that a model with one less class has a better fit (Collins and Lanza, 2009; Nylund et al., 2007; Nylund-Gibson and Choi, 2018). In addition, to avoid local solution convergence, we required the best log-likelihood value to be replicated for the solution selected for further analysis. The BCH approach in Mplus was used to inspect profile differences in academic achievement (Asparouhov and Muthén, 2014). We did not add gender to the LPA as a predictor to inspect gender differences in profile membership because of the considerable number of participants identifying themselves as “other” (*n =* 57, 4.8%) whom we opted to include in the analysis. Thus, gender differences were analyzed separately using logistic regression analyses with gender as the independent variable predicting the odds of belonging to one profile compared to others. Logistic regression analyses were conducted in SPSS 29.0.2.0.

## Results

**Figure 1:**
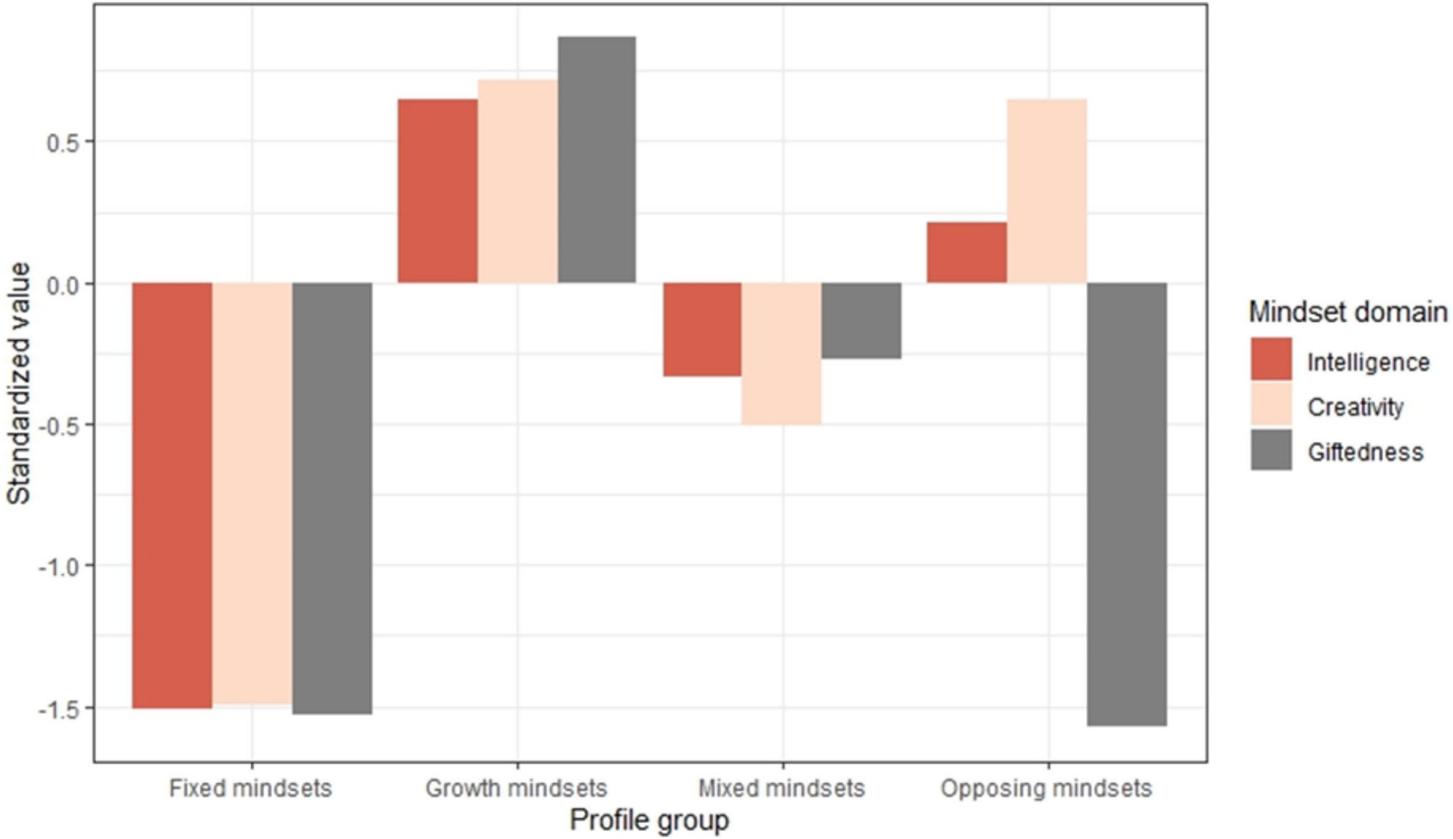
Standardized scores of the three mindset measures in the four latent mindset profiles.

Descriptives and bivariate correlations between all study variables are presented in Table 1. Based on CFA, a model with three correlated factors of intelligence, creativity mindset, and intelligence mindsets fit the data well, *χ*^2^(24) = 124.24 (*p* < 0.001);f CFI = 0.989, TLI = 0.983, RMSEA = 0.061, 90% C.I. (0.051, 0.072), SRMR = 0.022. Subsequently, LPA with the three mindset variables was conducted. Based on the fit indices of the LPA solutions (Table 2), the solution with four latent profiles was chosen for further analysis. Although the AIC and SABIC values decreased with additional profiles, solutions with more profiles resulted in lower entropy and extremely small profile groups. As for solutions with eight and nine profiles, the entropy increased and the SABIC values decreased notably from the seven-to the eight-profile solution (see also Supplementary Figure S1). However, for these solutions, multiple very small profile groups emerged (2–3% of cases) and, importantly, the best log-likelihood value was not replicated (Table 2). Therefore, the eight-and nine-profile solutions were discounted. The four-profile solution exhibited a high entropy and, compared to the three-profile solution, included an additional profile that clearly differed from other profile groups. The profiles were labeled as the following: *Fixed, Growth, Mixed*, and *Opposing Mindsets* (see Figure 1 and Table 3). The majority of students belonged to the *Growth Mindset* profile (44.4%), characterized by a high growth mindset on all mindset measures. The second largest profile group (37.07%) was the *Mixed Mindsets* profile, which was characterized by moderate levels of growth mindset on all measures. Slightly more than a 10th of the participants belonged to the *Fixed Mindsets* profile (11.85%), with a relatively fixed mindset regarding intelligence, creativity, and giftedness. The smallest profile group, which we labeled *Opposing Mindsets* (6.7%), was characterized by a relatively strong growth mindset about intelligence and creativity but a fixed mindset about giftedness.

**Table 1:** Descriptive statistics and correlations between all the measures.

Variable	Range	*M* (SD)	1	2	3	4	5	6	7	8	9
1. Intelligence Mindset	1–6	4.36 (1.23)	—								
2. Giftedness Mindset	1–6	4.35 (1.30)	0.570**	—							
3. Creativity Mindset	1–6	4.20 (1.43)	0.591**	552**	—						
4. Mathematics	4–10	8.51 (1.32)	0.126**	0.040	0.102**	—					
5. Reading	4–10	8.55 (1.12)	0.128**	0.084**	0.118**	0.703**	—				
6. Foreign languages	4–10	8.94 (1.06)	0.071*	0.021	0.081*	0.603**	0.611**	—			
7. Music	4–10	8.91 (0.87)	0.091*	0.131**	0.114**	0.421**	0.428**	0.304**	—		
8. Visual arts	4–10	8.64 (0.98)	0.139**	0.160**	0.111**	0.445**	0.525**	0.361**	0.388**	—	
9. Craft	4–10	8.50 (0.97)	0.145**	0.146**	0.147*	0.471**	0.507**	0.296**	0.423**	0.552**	—

*p < 0.05; **p < 0.01.

**Table 2:** Fit indices of the LPA solutions.

Nr. of profiles	Log-likelihood (LL)	Best LL replicated	Entropy	AIC	SABIC	VLMR
2	−5,175.25	Yes	0.731	10,370.504	10,388.827	<0.001
3	−5,043.847	Yes	0.739	10,115.694	10,141.346	0.002
4	−4,947.758	Yes	0.821	9,931.517	9,964.497	0.236
5	−4,903.579	Yes	0.791	9,851.158	9,891.468	0.198
6	−4,858.078	Yes	0.808	9,768.157	9,815.80	0.049
7	−4,828.145	Yes	0.773	9,716.290	9,771.257	0.374
8	−4,635.373	No	0.967	9,338.746	9,401.042	0.042
9	−4,602.520	No	0.952	9,281.039	9,350.665	0.148
10	−4,575.368	Yes	0.942	9,234.737	9,311.692	0.073

Solutions may not be trustworthy, when the best log-likelihood is not replicated.

**Table 3:** Descriptives of the mindset profiles.

Variable	Latent profile			
	Fixed mindsets (*n =* 131)	Growth mindsets (*n =* 491)	Mixed mindsets (*n =* 410)	Opposing mindsets (*n =* 74)
	M (SD) 95% CI [LL, UL]	M (SD) 95% CI [LL, UL]	M (SD) 95% CI [LL, UL]	M (SD) 95% CI [LL, UL]
Intelligence mindset	2.50 (0.99) [2.34, 2.68]	5.16 (0.78) [5.10, 5.24]	3.95 (0.89) [3.87, 4.04]	4.64 (1.12) [4.36, 4.88]
Creativity mindset	2.44 (1.02) [2.27, 2.63]	5.27 (0.72) [5.21, 5.33]	3.70 (0.81) [3.62, 3.78]	5.18 (0.80) [5.01, 5.35]
Giftedness mindset	2.02 (0.65) [1.90, 2.13]	5.44 (0.57) [5.39, 5.49]	3.82 (0.70) [3.75, 3.89]	1.96 (0.69) [1.82, 2.13]

### Between-profile differences in achievement

Based on omnibus Chi-square tests, the grades of students from the four mindset profiles differed in all the subjects we investigated (Table 4). Post-hoc pairwise comparisons indicated that students in the *Fixed-Mindsets* profile tended to have lower grades than students in the other profiles in all subjects (Table 4) apart from reading, foreign languages, and music, where *Mixed-Mindset* students achieved equally low grades. Additionally, compared to the *Mixed Mindsets* profile, the *Growth-Mindset* students achieved better grades in all subjects except math and foreign languages. Interestingly, the *Opposing-Mindsets* profile exhibited better grades than the other profiles in math and foreign languages. Regardless, students in this profile did not differ from students in the *Growth-Mindsets* profile in terms of their grades in the other subjects.

**Table 4:** Means and between-group differences in grades in all inspected subjects.

Variable	Latent profile				
	Fixed mindsets (*n* = 131)	Growth mindsets (*n* = 491)	Mixed mindsets (*n* = 410)	Opposing mindsets (*n* = 74)	χ^2^
	*M* (SE)	*M* (SE)	*M* (SE)	*M* (SE)	
Math grade	8.00^a^ (0.19)	8.59^b^ (0.10)	8.47^b^ (0.11)	9.20^c^ (0.12)	47.77***
Reading grade	8.24^a^ (0.18)	8.69^b^ (0.10)	8.42^a^ (0.14)	8.92^b^ (0.16)	21.19***
Foreign languages grade	8.82^a^ (0.12)	9.04^b^ (0.08)	8.91^a,b^ (0.12)	9.52^c^ (0.09)	46.97***
Arts grade	8.29^a^ (0.15)	8.78^b^ (0.06)	8.61^c^ (0.12)	8.60^b,c^ (0.12)	15.74**
Music grade	8.63^a^ (0.11)	9.03^b^ (0.07)	8.84^a,c^ (0.07)	9.00^b,c^ (0.12)	13.66**
Crafts grade	8.12^a^ (0.15)	8.66^b^ (0.07)	8.40^c^ (0.11)	8.73^b^ (0.13)	27.48***

Chi-square omnibus tests with 3 degrees of freedom. ***p < 0.001; **p < 0.01; *p < 0.05. Means with the same superscripts do not differ between profile groups.

### Gender composition

Logistic regression indicated that when using boys as the reference group, the *Fixed-Mindsets* (41% girls; *OR* = 0.39, *SE* = 0.08), *Opposing-Mindsets* (28% girls; *OR* = 0.23, *SE* = 0.07), and *Mixed-Mindsets* (47% girls; *OR* = 0.54, *SE* = 0.08) profiles contained significantly fewer girls than the *Growth-Mindsets* profile (61% girls; *p*s < 0.001). Additionally, compared to the *Mixed-Mindsets* profile, there were significantly fewer girls in the *Opposing-Mindsets* profile (*OR* = 0.43, *SE* = 0.12, *p* = 0.002). No other significant differences in gender distribution were found (*p*s > 0.10).

## Discussion

Our study aimed to understand lower-secondary school students views about the malleable or static nature of intelligence, giftedness, and creativity, and what kinds of mindsets profiles groups can be identified across the domains. Research on mindsets has predominantly focused on fixed and growth mindsets within individual domains. However, to understand both the general and domain-specificity of mindsets, it is crucial to investigate multiple mindsets across domains, as beliefs about the malleability of one attribute do not automatically apply across all domains (O’Keefe et al., 2018). Examining multiple domains at once can also help clarify the validity of the assumption that mindsets specific to a certain domain are most relevant to the outcomes associated with that domain (Lewis et al., 2021). In this study, our goal was to examine cross-domain mindset profiles. We employed a person-centered approach to identify mindset profiles with latent profile analysis and to examine how profile-group membership related to academic achievement as well as whether this membership differed by gender. *Growth, Fixed, Mixed*, and *Opposing* profiles were revealed, and these profiles were associated with differences in academic achievement. The results suggest that adolescent students’ learning-related mindsets were largely consistent across the three domains under investigation, although some students showed notable differences in their mindsets between the domains. In addition, membership of the profiles differed by gender, as girls were more likely to belong to the *Growth-Mindsets* profile across domains. Our discussion focuses on the profiles identified in this study and the relationship between profile-group membership, academic achievement, and gender.

### Mindsets profiles

We identified four mindset profiles: *Growth, Fixed, Mixed, and Opposing Mindsets*. Three of these profiles (*Growth, Fixed, and Mixed*) were consistent across the domains of intelligence, giftedness, and creativity. By contrast, one profile (*Opposing Mindsets*) was characterized by a growth mindset about intelligence and creativity but a fixed mindset about giftedness. The largest profile group (44.4%) consisted of students with a strong growth mindset in all three domains; thus, it was labeled *Growth Mindsets*. Identifying a clear growth-mindset profile was unsurprising, as previous studies conducted in the Finnish context have shown that many students tend to hold a growth mindset, particularly regarding intelligence (Kuusisto et al., 2017; Laurell et al., 2022).

In the second largest profile group (37.1%), students demonstrated moderate growth mindsets across domains. Dweck and Molden (2017) note that approximately 20% of students can exhibit an undecided mindset while other estimates suggest that the amount can be anywhere between 15 and 37% of the population (Kaijanaho and Tirronen, 2018).

The third profile group, with 11.9% of students, was characterized by a relatively fixed mindset in all domains; thus, the profile was named *Fixed Mindsets*. The smallest (6.7%), atypical profile—*Opposing Mindsets*—consisted of students who held a growth mindset in the domains of intelligence and creativity but a fixed mindset in the domain of giftedness. In this profile, students also performed exceptionally well in mathematics and languages. This profile aligns with the findings of Makel et al. (2015), Kuusisto et al. (2017), and Laurell et al. (2022), which have demonstrated that when comparing mindsets about intelligence and giftedness among school students, the domain of giftedness is often perceived as more fixed in nature, even in different cultural contexts (USA vs. Finland). However, as previously highlighted, it is suggested that a connotation in the word ‘gift’ implies that giftedness is obtained at birth. In sum, giftedness is perceived as more fixed in nature (Dweck, 2008). This is accurate, especially in languages (e.g., English and Finnish) where the word giftedness implies something given to a person without effort on the part of the recipient. More specifically, in the same way as in English, the Finnish words *lahjakas* and *lahjakkuus*, which can be translated directly as gifted and giftedness, are derived from the word *lahja*, meaning gift or talent in Finnish. Moreover, in everyday speech, and in the school context, the Finnish words describing giftedness/talent are likely often associated with high-achieving students. Further research, for example, using qualitative methods, is needed to understand what could explain the domain-specific variance in the implicit beliefs about intelligence, giftedness, and creativity within this student group.

When simultaneously measuring multiple domains, we found moderate correlations between the mindsets. This suggests that mindsets about intelligence, giftedness, and creativity exhibit a degree of consistency, reinforcing the notion of generalized beliefs regarding growth or fixed ideas about human attributes, as was noted by Lewis et al. (2021). Notwithstanding, the results also suggest an *Opposing-Mindsets* profile, including both domain-general and domain-specific views. These findings align with research conducted by Petscher et al. (2017), Schroder et al. (2016), and Puusepp et al. (2023). Furthermore, these results mirror those of Lewis et al. (2021), who found evidence that beliefs across domains consist of a common global (or general) mindset belief plus, in some circumstances, domain-specific mindsets. They also demonstrated at least some domain-specific aspects of mindsets across multiple domains. In sum, these results suggest that students’ perceptions of intelligence and creativity are more similar than their perceptions of the malleability of giftedness.

### Mindsets profiles and academic achievement among the groups

When we compared the academic achievement of the *Growth*, *Mixed, Fixed*, and *Opposing-Mindsets* profile groups in mathematics, reading, 1st compulsory foreign language, music, visual arts, and crafts, we found that students’ academic grades differed according to their profile While the *Growth-Mindsets* profile appeared to be the group with the highest overall academic grades, surprisingly, the best grades in math and languages were found among the profile with *Opposing-Mindsets*. Our results partially align with previous variable-oriented studies in the sense that growth-mindset students outperformed those with a fixed mindset (e.g., Claro et al., 2016). However, in our findings, students from the *Growth Mindsets* and *Mixed Mindset*s profiles did not differ in their math grades, although it has been shown that differences in achievement according to mindset are most prominent in mathematics (Gunderson et al., 2018).

Nevertheless, similar results about students’ mindsets in the domains of intelligence and giftedness were obtained in a variable-oriented study by Kuusisto et al. (2017), which was also conducted in the context of a Finnish school. They found that students’ growth-oriented views about intelligence but fixed ideas about giftedness were associated with higher grades in mathematics. Interestingly, the *Opposing-Mindsets* group also outperformed the other profile groups in their foreign language grades but not, for example, in reading, which has been commonly a subject related to proficiency in mathematics (Koponen et al., 2020). Nevertheless, in the context of Finnish education, it seems that the domain of giftedness is a somewhat loaded construct, and, for a minority of students, fixed mindset beliefs are related to high performance in mathematics. In Finland, in lay speech, it remains rather common to describe someone as a “math person,” which refers to the idea that some individuals possess an innate ability in math while others simply do not. Such expressions are likely to reinforce the belief that high performance in math is related to individuals’ giftedness or natural talent above anything else. These thoughts might (unconsciously) affect, in particular, students who show natural interest and high ability in mathematics from an early age.

Such students probably receive praise from parents, peers, and teachers for their apparent talent in math, and they easily gain high grades in the subject at school. Nonetheless, such praise can be harmful and may prevent these students from reaching their full potential: Dweck (2007) has noted in relation to intelligence that students with a fixed mindset tend to emphasize “looking smart.” Consequently, they may be unwilling to show vulnerability when facing challenges or failures, which may lead to avoidance of challenging learning opportunities. The same may be true of fixed ideas of giftedness, and students with an *Opposing-Mindsets* profile might hold such notions especially in relation to math (Gunderson et al., 2018). Consequently, in the long run, these students might not be able to exploit their full capability in specific areas despite their talent (Burnette et al., 2022; Dweck, 2007).

However, it is important to note that Finnish education legislation does not explicitly address gifted students or recognize them as a subgroup with special needs (Laine and Tirri, 2021). This lack of recognition can prevent high-performing students from fulfilling their potential or receiving the necessary support, as the Finnish educational system employs a differentiation approach aimed at identifying gaps between students’ knowledge and the curriculum content (Laine and Tirri, 2021). Additionally, high-performing students may not be sufficiently challenged, as this depends on the individual efforts of teachers. While these (*Opposing Mindset*) students perform well in mathematics in lower secondary school (as seen in this study), it is possible that if they proceed to study STEM-related subjects in higher education, they might encounter challenges as the materials become more complex and demanding. This possible threat should be acknowledged. There is an elevated risk of dropping out from the studies if these individuals are not able to change their implicit beliefs about giftedness. Nevertheless, this is dependent on the development of students’ mindsets, as some people might retain their fixed mindsets throughout their life course while others might abandon such views as they grow older.

On the other hand, for Opposing-Mindsets students, a fixed mindset about giftedness might also reflect their self-assurance about their skills. A previous study found that primary and upper secondary school students’ implicit beliefs about intelligence did not induce higher grades in math or languages; instead, students’ previous school achievements affected their mindset beliefs, and this was mediated by perceptions of their academic competence (Leondari and Gialamas, 2002). However, it is notable that although the number of students in the *Opposing-Mindsets* group was small compared to the whole sample in our study, it is possible that one or two students in each class hold such a mindset. However, most students we investigated held a growth mindset about giftedness, which underlines that not all individuals automatically develop fixed beliefs about giftedness. Furthermore, it is possible that those students who rated giftedness differently to intelligence and creativity held different conceptions of giftedness than students in the other profiles. This could be revealed by future studies through qualitative research using interviews to grasp underlying factors such as family background, and other relevant factors.

When we further compared the profiles and focused on mixed mindsets and growth mindsets, we discovered that the *Growth-Mindsets* profile outperformed the *Mixed-Mindsets* profile in all other subjects than math and foreign languages. Moreover, the *Growth Mindsets* profile outperformed the other profile groups in most of the subjects in addition to reading. As already mentioned in reference to a study by Leondari and Gialamas (2002), could it be that previous school achievements affect the mindsets of these students rather than vice versa?

In terms of *Fixed Mindsets*, we discovered that students in this profile achieved lower grades than students within the *Growth-Mindsets* profile in all other subjects than reading and music. Based on mindset theory and the findings of multiple variable-centered studies (e.g., Blackwell et al., 2007; Burnette et al., 2023), this result is unsurprising, but it also indicates that growth-mindset beliefs are not always associated with higher grades or performance in all subjects. Academic achievement also differed between the profiles of *Fixed* and *Mixed Mindsets* as the grades of students with a *Fixed Mindsets* profile were lower than those of students in the *Mixed Mindset*s profile in every other subject than reading, foreign languages, and music. Nevertheless, this finding also underscores the importance of investigating both the general and subject-specific aspects of mindsets as even if individuals exhibit a general mindset, they might also hold subject-specific mindsets in areas such as math (Puusepp et al., 2023) and language learning (Petscher et al., 2017).

### Mindsets profiles and differences between genders

Gender differences have been found to be rather common in motivational studies (Butler, 2014); however, mindset research is more ambiguous in its findings on gender differences, as such differences may only become apparent when studies include subject-domain-specific mindsets (e.g., math) alongside more general mindsets (Yu and McLellan, 2020). We decided to investigate girls’ and boys’ membership of the different mindset profiles because there are clear gender differences in academic achievement in compulsory education in Finland (Hautamäki et al., 2013, 2015; Finnish National Agency for Education, 2014). Moreover, previous Finnish mindsets studies have observed gender differences, with boys more likely to hold fixed ideas about intelligence and giftedness (Kuusisto et al., 2017; Laurell et al., 2022).

We used boys as the reference group and discovered that in the *Fixed, Mixed,* and *Opposing* profiles, there were noticeably fewer girls than in the *Growth-Mindsets* profile. Additionally, when the *Mixed-Mindsets* and *Opposing-Mindsets* profiles were investigated, the *Opposing-Mindsets* profile included significantly fewer girls. As there were more boys in this profile, which included fixed mindsets about giftedness, the findings align with previous Finnish studies (Kuusisto et al., 2017; Laurell et al., 2022), which also found that adolescent boys were more likely than their female counterparts to hold fixed mindsets about giftedness while no gender differences were observed in the domain of intelligence.

We found that girls were overrepresented in the *Growth* and *Mixed-Mindsets* profiles. This result aligns with a previous study which found that boys tended to prioritize validating their competences or avoiding displays of incompetence (i.e., a performance approach and avoidance goals; Yu and McLellan, 2020) while girls were overrepresented in profiles with dominant mastery goals. In other words, girls are more likely than boys to exhibit a willingness to develop their skills; thus, they are more likely to develop a growth mindset. One explanation for girls’ superior grades at school in general is the greater effort that they put into their studies (Butler and Hasenfratz, 2017), which is a core behavior linked to holding a growth mindset. Relatedly, the overrepresentation of girls in the *Growth* or *Mixed-Mindsets* profiles in our study might help explain boys’ poorer-than-average performance in the school system in Finland (OECD, 2019). This suggestion aligns with the results from a global meta-analysis performed by Lindberg et al. (2010) and a national analysis in Finland conducted by Metsämuuronen and Nousiainen (2021), which both suggested that while average mathematics performance between genders is quite similar, boys are more likely to be represented at both the high and low ends of the performance spectrum.

### Limitations and future research

Our study contains several limitations that should be considered and addressed in future research. Our study explored students in Finnish lower-secondary school, which limits the generalizability of the findings to other cultural or educational contexts. Furthermore, our study relied on self-reported questionnaires to measure students’ mindsets—their views about the malleability of characteristics—which may have introduced biases, such as misinterpretation of questions, failure to take the questions seriously, or deliberately choosing not to answer. Additionally, participants’ preconceptions about the nature of intelligence, giftedness, and creativity may have influenced their responses. However, the mindset research is interested on people’s conceptions of attributes as developmental of trait-like, not on understanding how individuals themselves define the constructs. Still, this issue relates to the context present as different cultural norms, or prior exposure to discussions about giftedness does influence on the ideas and perceptions students have. Future research could, thus, include qualitative methods, such as interviews or open-ended survey items, to explore how students conceptualize giftedness. This would provide additional context for understanding mindset profiles, particularly the fixed giftedness mindset seen in the Opposing Mindsets group.

It is also important to note that the mindset scale used for data collection only included entity items (Dweck, 2008); thus, it does not necessarily capture the nature of the students’ mindsets as thoroughly, as the recommendation to omit the incremental items assumes that entity and incremental views represent two polar theories (Combette and Kelemen, 2024). Moreover, it should also be noted that some more recent studies (Dupeyrat and Mariné, 2005; Scherer and Campos, 2022) have questioned whether the implicit intelligence theory construct is unidimensional (see, for example, Combette and Kelemen, 2024; Lüftenegger and Chen, 2017). Moreover, we did not account for broader motivational constructs such as effort beliefs or achievement goals, which could have provided a more thorough understanding of this complex phenomenon. Profiling students based on a broader set of motivational variables, rather than an implicit theory of intelligence scale alone, could have revealed more in-depth information about the students’ mindsets and how other motivational constructs were related to them in the creation of “meaning systems.”

Furthermore, it is highly relevant to consider how mindsets are measured in future studies and what can be claimed based on data gathered with mindset items alone. Moreover, while our latent profile analysis identified four profiles (*Fixed*, *Growth*, *Mixed*, and *Opposing Mindsets*), the smallest profile (*Opposing Mindsets*) comprised only 6.7% of the sample, which may reduce the reliability of conclusions drawn about this specific profile. The present study relied solely on academic grades, which may not fully capture student performance and skill complexities. Grades are subjective and reliant on teachers’ evaluations; thus, they may vary from student to student for several reasons. Therefore, academic grades may not entirely reflect students’ potential across all areas of learning.

Finally, we used a cross-sectional design, which limited our ability to infer causality between mindsets and academic achievement. In future studies, it is crucial to use longitudinal data to assess how mindsets evolve over time and whether students remain in the same profile groups or how stable the profiles are during the lower-secondary school years. In addition, it is important to examine how profile group membership influences academic outcomes among students. Future studies should also investigate broader motivational constructs such as effort beliefs and achievement goals. Additionally, it would be beneficial to include scales with global mindset beliefs and domain-specific mindsets (e.g., Lewis et al., 2021). To be able to observe the domain-specificity and generality of students’ mindsets more reliably, it seems necessary to investigate mindsets further from this perspective.

## Conclusion

In conclusion, the results of the current study suggest that mindsets in the domains of intelligence, giftedness, and creativity form distinct profiles among adolescent students in lower secondary schools, with profile membership linked to academic achievement and gender. The study also highlights the value of a person-centered approach when examining mindsets across multiple general domains. Latent profile analysis provided an opportunity to identify hidden patterns in individual students’ general mindsets and specifically illustrated how the profile groups differed between subjects and gender. We identified four mindset profiles across the three mindset domains using this method—*Growth, Fixed, Mixed*, and *Opposing Mindsets—*and differences were found in achievement in various subjects related to each profile group. Our results align with previous studies highlighting the intricacy of students’ mindset beliefs. Our findings show that mindset beliefs are highly relevant in the school context, as they can affect achievement in specific subjects. However, our results emphasize that even generalized mindsets do not uniformly affect academic achievement across all subjects. Rather, the findings were more nuanced, with notable differences between subjects with different orientations and goals. Although students with growth mindsets generally performed extremely well across a range of academic subjects, interestingly they were outperformed in math by students with a fixed mindset about giftedness—a unique combination of growth and fixed beliefs that warrants further investigation, as do gender differences within and across mindset domains. Moreover, our study emphasizes the importance of simultaneously examining mindset beliefs across multiple domains. Educators should not assume that adolescent learners neatly fit into growth or fixed mindset categories, as some may hold more complex beliefs. By contrast, others may hold generalized views on their attributes and abilities. Thus, it is necessary to explicitly identify students’ profiles to support students with varying mindsets and beliefs instead of simply assuming that academic achievement provides the necessary motivation for them to continue their ability development or fulfill their potential. Although our findings suggest that domain specificity matters, it remains unclear how mindsets about intelligence, giftedness, and creativity manifest in the everyday life of schools. Consequently, further research in the Finnish context is necessary on domain-specificity and the generality of mindsets, particularly intelligence, giftedness, and creativity.

## Data Availability

The authors will make the raw data supporting this article’s conclusions available to any qualified researcher without undue reservation.

## Appendix

**Table A1:** *Post hoc* pairwise comparison significance levels and effect sizes for grades.

	1	2	3
Math grade			
1. *Fixed Mindsets*			
2. *Growth Mindsets*	0.39***		
3. *Mixed Mindsets*	0.29*	ns	
4. *Opposing Mindsets*	0.80***	0.41***	0.45*****
Reading (Finnish/Swedish) grade			
1. *Fixed Mindsets*			
2. *Growth Mindsets*	0.36**		
3. *Mixed Mindsets*	ns	0.20***	
4. *Opposing Mindsets*	0.51***	ns	0.35****
Foreign languages grade			
1. *Fixed Mindsets*			
2. *Growth Mindsets*	*		
3. *Mixed Mindsets*	ns	ns	
4. *Opposing Mindsets*	***	***	*****
Arts grade			
1. *Fixed Mindsets*			
2. *Growth Mindsets*	***		
3. *Mixed Mindsets*	*	***	
4. *Opposing Mindsets*	*	ns	ns
Music grade			
1. *Fixed Mindsets*			
2. *Growth Mindsets*	*****		
3. *Mixed Mindsets*	ns	***	ns
4. *Opposing Mindsets*	***	ns	ns
Crafts grade			
1. *Fixed Mindsets*			
2. *Growth Mindsets*	***		
3. *Mixed Mindsets*	*	****	
4. *Opposing Mindsets*	*****	ns	***

*p* < 0.001; ***p* < 0.01; **p* < 0.05; ns, non-significant.
