# Supplementary figures and images for: Students’ cross-domain mindset profiles and academic achievement in Finnish lower-secondary education
Source: Front Psychol. 2025 Jan 23;16:1514879. doi: 10.3389/fpsyg.2025.1514879 (PMC11800590; doi:10.3389/fpsyg.2025.1514879)

**Figure S1**

*Elbow plot of SABIC scores*

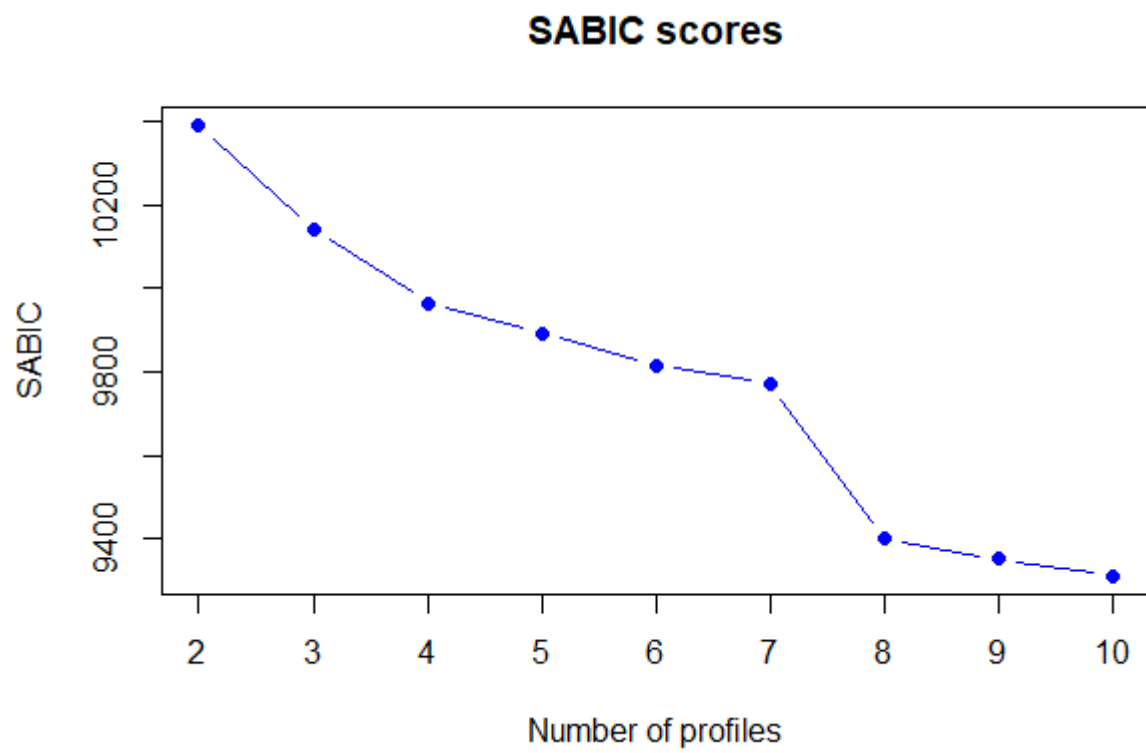

Supplement: Supplementary file 1 [file Image_1.pdf]
